# Supplementary material for: Community participation enhanced prenatal mental health through strengthening peers’ support and partners’ communication in Chinese mothers: A cross-sectional study
Source: PLoS One. 2025 Oct 6;20(10):e0333415. doi: 10.1371/journal.pone.0333415 (PMC12500102; doi:10.1371/journal.pone.0333415)
Supplement: Attachment — Submitted filename: Response to Reviewers 1.docx [file pone.0333415.s002.docx]

Dear reviewers

Thank you for your thoughtful and constructive feedback. We sincerely appreciate the time and effort you devoted to reviewing our manuscript. Your insightful comments have been invaluable in helping us enhance the clarity, structure, and academic quality of our work.

In response to your suggestions, we have carefully revised the manuscript. Minor changes are documented in the point-by-point response table below, with each revision clearly explained. To facilitate your review, we have also highlighted all modifications in the revised manuscript. Where confusion may have arisen, we have added clarifying explanations to better communicate our research aims, methodological decisions, and interpretations of the findings. The manuscript you received contains two documents: the Manuscript and the Revised Manuscript with Track Changes. According to the order established by PLOS ONE journals, the Manuscript is presented before the Revised Manuscript with Track Changes. To facilitate your review, the number of lines referenced in our response corresponds to the number of lines in the Manuscript. We hope these clarifications will help you gain a clearer understanding of our study and its contributions.

Once again, we sincerely thank you for your time, attention, and generous input. Your guidance has played a crucial role in improving this manuscript, and we are deeply grateful for the opportunity to revise and resubmit it.

Sincerely,

The authors

|  | **Comments** | **Response** |
| --- | --- | --- |
| **Reviewer 1** | Clarify which 'father' you are referring to in this context: "Research indicates that increased emotional support from fathers…" .Is it the father of the pregnant woman or father of the unborn baby? | We appreciate your insightful observation regarding the potential ambiguity associated with the term 'fathers.' We recognize that the original wording lacked precision, which could lead to misunderstandings. In this study, all references to 'fathers' were intended to refer specifically to the partners of the pregnant women—that is, the fathers of the unborn children. To improve clarity and maintain professional accuracy, we have systematically revised the terminology throughout the manuscript. Specifically, the term used in line 98 has been changed to 'partner' to adopt a more inclusive and precise expression, while the term in line 358 has been modified to 'fathers of unborn children' to explicitly specify the intended referent. These revisions aim to ensure consistency and eliminate any ambiguity in the text (see line 115 and line 415). |
|  | Some statements in the paper should be backed with appropriate references. | We appreciate your recognition of the value of our research and your observation regarding the inadequacy of references for several claims in the manuscript. We wholeheartedly agree that high-quality academic writing must be firmly rooted in robust and well-documented evidence. In response to your suggestion, we have meticulously reviewed the entire manuscript, paying particular attention to statements that were previously unsupported by citations. We have now enriched these sections with multiple authoritative sources from both domestic and international literature, thereby enhancing the rigor and credibility of our arguments (see lines 40–62, 65–68, 99--100, and 401–406). |
|  | Authors should get the services of language editor as some grammatical errors are noticeable in the paper such as:  i. The relationship between a mother and her couple…  ii. …communication and splays mediating roles…  iii. The mediating effect test reveals that peers’ support support… | Thank you for your rigorous attention to linguistic details. We sincerely apologize for the grammatical and lexical issues identified in the manuscript. In response to your comments, we have carefully revised each of the issues you pointed out and conducted a thorough language review of the entire manuscript to improve the clarity and professionalism of our academic writing. The specific revisions are as follows:  1. The original sentence lacked clarity. It has been revised to: "The relationship between a woman and her partner is frequently regarded as a more significant protective factor in close relationships than those with parents and friends." (refer to lines 109–111)  2. The original wording was inappropriate. It has been revised to: "Support from peers, whether facilitated through offline groups or social media platforms, serves as a mediating factor in the relationship between community participation and PMH." ( refer to 167–169)  3. The original sentence contained a redundant repetition ('support support'). It has been corrected to: "The mediating effect test demonstrates that peer support and partner communication serve as mediators in the relationship between community participation and PMH." ( refer to lines 397–399) |
| **Reviewer 2** | All the mothers in this study are still in gestation which would be considered prenatal, as the title suggests. However, throughout the paper, the word perinatal is used which usually includes mothers for at least one year after birth. For consistency, stick to using “prenatal” except for where past research and literature focused on the wider perinatal period. | Thank you for your valuable feedback regarding the inconsistency in terminology usage. We fully concur with your observation that maintaining terminological consistency and scientific accuracy is crucial when describing the study population and research scope. As you rightly pointed out, all mothers who participated in this study were between 13 and 41 weeks of gestation, which falls within the prenatal period. To ensure that our terminology accurately reflects the study population and to prevent any potential confusion for readers, we have systematically reviewed the manuscript. We have replaced all non-referenced uses of the term 'perinatal' with 'prenatal', including in the title and throughout the main text. In instances where the original wording in cited literature uses 'perinatal' while the study content clearly includes the prenatal stage, we have retained the term but added a clarifying note to maintain academic precision. |
|  | The first paragraph lines 38 to 50 do not tell the reader directly what the paper is about. If this study is focusing on non-pathological experiential physical and mental imbalances in prenatal mental health (PMH) (as stated in the last sentence), then mentioning prenatal depression and fear of childbirth could confuse the reader unless these are included in your PMH measure. | Thank you for highlighting the issue of insufficient clarity in the logical structure of the introduction. In response to your suggestion, we have restructured the opening paragraph to more clearly define the focus of this study. We begin by introducing a dual-dimensional framework of mental health, which distinguishes between pathological imbalance and non-pathological experiential imbalance. We emphasize that the latter is particularly prevalent during the prenatal period, especially in Asian countries such as China, where it is closely tied to sociocultural contexts. While we acknowledge the importance of issues such as prenatal depression and fear of childbirth, we clarify that this study focuses specifically on the non-pathological, subjectively experienced forms of psychological disequilibrium (refer to lines 42–65, and references 5–6). |
|  | Furthermore, the paper is focusing on social support and community participation which is the novel aspect of the paper and should be mentioned as early as possible at the end of this paragraph. | Thank you for your insightful suggestion. As you rightly noted, the key innovation of this study lies in the incorporation of social support and community participation into the analytical framework for understanding prenatal mental health. In the revised manuscript, we have added a statement at the end of the first paragraph to emphasize how social support and community participation serve as moderating and mediating variables that influence the psychological well-being of pregnant women. This addition establishes a conceptual foundation for the subsequent analysis and discussion (refer to lines 53–62,and references 7–11 ). |
|  | The opening paragraph also mentions cultural factors (lines 47 – 49), which although interesting, would be best moved to the beginning of the paragraph at line 62 when highlighting the Chinese context. This would make it clearer that the current paper does not address cultural factors listed but does consider culture in a novel way (for example, ChaxuGeju). | Thank you for your constructive feedback regarding the text structure. Following your suggestion, we have relocated the discussion on cultural factors from the original lines 47–49 to a new position at lines 75–77. |
|  | In line 180, it is stated that 1705 responses were “validly answered” but no criteria of what was considered a valid answer is stated. Please state the criteria that deemed a response usable. | Thank you for your insightful comments regarding the imprecise description of the questionnaire data. In response to your suggestion, we have clarified the two criteria for classifying a questionnaire response as 'valid' in lines 203–208: (1) the questionnaire must be fully completed, with no missing items; and (2) the respondent must not display obvious logical inconsistencies or provide deliberate erroneous answers. Furthermore, we have included relevant literature to underscore the significance of questionnaire validity in ensuring the reliability of survey results (refer to lines 203–208,and references 36–37). |
|  | For the section on “social support from peers” (line 207), it is unclear what were the social support categories and what items were given for each of the types of support. Line 209 states “such as” which implies that more types of social support were given then the two listed. It is best to state the total amount of social supports or if it was just these two, make this clear. In addition, instrumental support is an academic term not always understood by non-experts (at least in English) so how was instrumental support and the other types of support defined to participants? | Thank you for your valuable suggestions regarding the classification and expression of social support. In light of your advice, we have revised the section on "peer social support" as follows. First, this study categorizes social support into three functional types: emotional support, instrumental support, and informational support, encompassing a total of 16 measurement items that cover specific supportive contents within each category. Second, to enhance readability, we have provided concise definitions for each type of support, drawing from the classic framework established by Rodriguez & Cohen (1998). Instrumental support refers to tangible assistance that individuals receive, such as financial aid or help with daily tasks. Informational support involves the provision of relevant knowledge, advice, or guidance aimed at helping individuals cope with challenges or make informed decisions. Emotional support includes expressions of empathy, care, reassurance, and trust, providing individuals with opportunities for emotional expression and psychological relief (see lines 241–253, reference 40). |
|  | For your mediation results, confidence intervals would be useful to have for the different effects. | Thank you for pointing out the shortcomings in the presentation of our results. In response, we have added the 95% confidence intervals for each major effect path in Table 3 to enhance the transparency and interpretability of our findings. (See lines 378–379) |
|  | In lines 400, it is claimed that the study “helped mothers in managing various types of support” which is too bold for the paper. The word manage implies some sort of researcher-led intervention or experiment to help mothers which is beyond the aims of this study. The study’s cross-sectional design has more helped our understanding of the importance of community participation and social support for mothers which may have implications for implementing and managing support. | Thank you for highlighting the potential for misunderstanding in our original wording. We fully agree that the cross-sectional design of this study does not allow for direct inference of intervention effects. Accordingly, we have revised the relevant statement to more accurately reflect the study’s aims and findings. The text now reads: “This study aims to examine the role of community involvement and social support in the lives of mothers, providing insights that may inform the design and implementation of supportive interventions.” (See lines 458–460) |
|  | More limitations should be considered. For example, the measure BMSWBI is more about subjective mental wellbeing rather than mental health. Therefore, symptoms of depression or anxiety participants mentioned in the introduction might not have been picked up on. | Thank you very much for your thoughtful and constructive feedback. We sincerely appreciate your careful review and fully acknowledge the valuable concerns you have raised. In response, we have revised the Limitations section of the manuscript accordingly (see lines 460–475) and would like to provide the following detailed responses. We acknowledge that the Body–Mind–Spirit Well-Being Inventory (BMSWBI) primarily captures subjective and culturally grounded dimensions of psychological well-being and may not comprehensively detect clinical manifestations of mental disorders such as depression or anxiety. As our study focused on experiential imbalance—a culturally nuanced conceptualization of mental distress rather than clinical diagnosis—the BMSWBI was selected for its alignment with our theoretical framework and its validated application in Asian populations. Nonetheless, we agree that the absence of standardized diagnostic tools such as the PHQ-9 or GAD-7 limits our ability to identify clinical symptomatology. This limitation is now explicitly acknowledged in the revised manuscript; alongside a recommendation that future research incorporate such instruments to further explore the intersections between culturally defined well-being and clinically defined mental health conditions. (See lines 460–475) |
|  | In addition, participants were not asked if their experience of different community organizations was positive or negative. We also do not know what type of support the participants felt they received from each organization. | Thank you very much for your thoughtful and constructive feedback. We sincerely appreciate your careful review and fully acknowledge the valuable concerns you have raised. In response, we have revised the Limitations section of the manuscript accordingly (see lines 460–475) and would like to provide the following detailed responses. We fully agree that subjective evaluations of experiences with community organizations—whether positive, neutral, or negative—are essential for understanding the impact of community participation on well-being. While our study measured participants’ involvement across various community groups, it did not capture their perceptions of the quality of those interactions, nor did it identify specific organizations or the distinct types of support they provided. This significant limitation has been explicitly acknowledged in the revised manuscript. Although we were unable to examine all types of organizations in detail, our data did assess participants’ perceived emotional, instrumental, and informational support from key domains such as family, healthcare, peer groups, community, government, and social media. These indicators offer a multidimensional understanding of social support that complements the broader analysis presented in our findings. The implications of these findings will also be reflected in other research outcomes. (See lines 460–475) |
|  | Your study also includes only Chinese prenatal mothers and so we do not know if the results are generalisable to mother’s one-year post-birth or mothers in other countries. | Thank you very much for your thoughtful and constructive comments. We sincerely appreciate your careful review and fully acknowledge the valuable concerns you have raised. In response, we have revised the Limitations section of the manuscript accordingly (see lines 460–475) and would like to provide the following detailed responses. We appreciate your observation regarding the demographic scope of our sample. Since our study focuses specifically on prenatal mothers in China, the findings may not be generalizable to postpartum mothers or to mothers in different cultural contexts. We have clarified this limitation in the manuscript. Additionally, we would like to highlight that a parallel longitudinal qualitative study is currently underway, tracking a subset of 83 participants from pregnancy through six months postpartum. This ongoing research will enable us to investigate the evolution of maternal well-being and support dynamics throughout the perinatal period. Future also includes cross-cultural comparisons to assess the consistency of these patterns across diverse sociocultural settings. (See lines 460–475) |
|  | Lines 92, 101, and 216, the word “couple” would be more natural as “partner” in these cases. | Thank you for your suggestion regarding the use of terminology. In response, we have revised the manuscript to replace “couple” with the more appropriate and inclusive term “partner” in all relevant instances. |
|  | Line 150, I think the hypothesis is meant to say “communication play mediating roles” | Thank you for your insightful observation. Based on your feedback, we have revised the hypothesis statement to more clearly reflect the mediating role of peer support. The updated sentence now reads: “Support from peers, whether facilitated through offline groups or social media platforms, serves as a mediating factor in the relationship between community participation and PMH.” (see lines 167–169) |
|  | The descriptive statistics section (Line 242) is not needed as it is covered by Table 1. Lines 243 – 245 are not needed as this is already covered in the participants section. Lines 245 – 247 can be added to the participants section. I would suggest starting the Results with the correlational analyses and mentioning the descriptives can be found in Table 1 within the section. | We appreciate your insightful and constructive suggestion. We fully concur with your observation that repeating descriptive statistics already presented in Table 1 within the Results section leads to unnecessary redundancy. In response to your recommendation, we have made the following revisions:  First, we have relocated the descriptive statistics originally presented in the Results section (line 242) to the 'Participants' subsection, thereby consolidating the presentation of sample characteristics. This restructuring enhances the clarity and logical coherence of the manuscript (see lines 211–216).  Second, following your advice, we have introduced a correlation analysis at the beginning of the 'Results' section to underscore the statistical relationships among the dependent, independent, and mediating variables. This addition improves the logical flow leading into the subsequent model testing (see lines 309-323).  Finally, we have explicitly indicated in the text that Table 1 contains the complete set of descriptive statistics to prevent redundancy. |
|  | At the beginning of the correlation section, it would be useful to remind readers what the hypotheses were so readers understand the purpose of these correlations. | Thank you very much for your valuable suggestion. We fully agree that restating the core hypotheses and key variables at the beginning of the correlation analysis section enhances readers’ understanding of the purpose and logical structure of the analysis. In response to your recommendation, we have revised the opening paragraph of the correlation analysis section to explicitly restate the main hypotheses of the study and clearly define the dependent, independent, and mediating variables. This revision improves the readability and coherence of the section, thereby establishing a stronger theoretical foundation for the subsequent structural equation modeling and path analysis (see lines 285–294). |
|  | In addition, you mention when things (partially) support H1, H3, and H4 but do not mention H2 directly in the results. | Thank you for bringing to our attention the omission of a response regarding H2 in our article; this was indeed an oversight on our part. We have now included a comprehensive response to H2 in the results section, specifically on line 349. |
| **Journal Requirements** | Please ensure that your manuscript meets PLOS ONE's style requirements, including those for file naming. | Thank you for your thorough review and valuable guidance regarding the manuscript's adherence to PLOS ONE's formatting and ethical standards. We have meticulously addressed each of your points as follows:  1. We have revised the manuscript and all accompanying files to ensure compliance with PLOS ONE's formatting and file naming requirements. |
|  | Please ensure that you have an ORCID iD and that it is validated in Editorial Manager. To do this, go to ‘Update my Information’ (in the upper left-hand corner of the main menu), and click on the Fetch/Validate link next to the ORCID field. This will take you to the ORCID site and allow you to create a new iD or authenticate a pre-existing iD in Editorial Manager. | 2. The corresponding author has successfully registered and validated their ORCID id in the Editorial Manager system, following the provided instructions. |
|  | Please include your full ethics statement in the ‘Methods’ section of your manuscript file. In your statement, please include the full name of the IRB or ethics committee who approved or waived your study, as well as whether or not you obtained informed written or verbal consent. If consent was waived for your study, please include this information in your statement as well. | 3. We have added a comprehensive ethics statement in the Methods section of the revised manuscript. This now includes the full name of the ethics committee that approved our study, the approval reference number, and a clear explanation of the informed consent process. (see revised Methods section, lines 196–201). |
|  | All PLOS journals now require all data underlying the findings described in their manuscript to be freely available to other researchers, either 1. In a public repository, 2. Within the manuscript itself, or 3. Uploaded as supplementary information. | 4. In accordance with PLOS ONE's data sharing policy, we have made all data underlying the findings of this study available within the manuscript itself. (see lines 507-509) |
|  | Please remove all personal information, ensure that the data shared are in accordance with participant consent, and re-upload a fully anonymized data set. | 5. Personal identifiers have been removed to ensure participant confidentiality, and all shared data comply with the terms of informed consent obtained from participants. |
|  | Please include captions for your Supporting Information files at the end of your manuscript, and update any in-text citations to match accordingly. | 6. All Supporting Information files have now been properly captioned and listed at the end of the manuscript. In-text citations referencing these files have also been updated for consistency and clarity (see lines 503–509). |
|  | Please review your reference list to ensure that it is complete and correct. | 7. We have thoroughly reviewed the reference list to ensure that all citations are complete, accurate, and formatted according to PLOS ONE style. Missing or incomplete entries have been corrected (see lines 511–681). |
