# Supplementary material for: Community participation enhanced prenatal mental health through strengthening peers’ support and partners’ communication in Chinese mothers: A cross-sectional study
Source: PLoS One. 2025 Oct 6;20(10):e0333415. doi: 10.1371/journal.pone.0333415 (PMC12500102; doi:10.1371/journal.pone.0333415)
Supplement: Attachment — Submitted filename: Response to Reviewers 0805.docx [file pone.0333415.s003.docx]

Dear reviewers

Thank you for your thoughtful and constructive comments. We sincerely appreciate the time and effort you have devoted to reviewing our manuscript. Your insightful comments have been invaluable in helping us enhance the clarity, structure, and academic quality of our manuscript.

We have carefully revised the manuscript in response to your suggestion. Minor changes have been documented in the point-by-point response table below, with each revision clearly explained. To facilitate your review, we have highlighted all modifications in the revised manuscript. Where confusion may have arisen, we added clarifying explanations to better communicate our research aims, methodological decisions, and interpretations of the findings. The manuscript you have received contains two documents: the Manuscript and the Revised Manuscript with Track Changes. According to the order established by the PLOS ONE journals, the manuscript is presented before the Revised Manuscript with Track Changes. To facilitate your review, the number of lines referenced in our responses corresponds to the number of lines in the manuscript. We hope that these clarifications will help you to gain a clearer understanding of our study and its contributions.

Once again, we sincerely thank you for your time, attention and generous input. Your guidance has played a crucial role in improving this manuscript and we are deeply grateful for the opportunity to revise and resubmit it.

Sincerely,

The authors

|  | **Comments** | **Response** |
| --- | --- | --- |
| **Reviewer 1** | 1. The abstract states that 'a cross-sectional survey was conducted…' but no mention of this in the full text. | Thank you for highlighting the deficiencies in our report results. In response, we have incorporated the language related to the cross-sectional survey into the methodology and limitations of the research design.  In the methods section, we added the following statement: "We conducted a cross-sectional survey on a representative sample of pregnant women in China". (see lines 181-182)  Additionally, in the limitations section, we included "the COVID-19 quarantine policy has significantly hindered our ability to collect cross-regional and large-scale data. Consequently, this study utilized regional samples and gathered data in clinical settings, which may limit our capacity to observe the evolution of mental health and support throughout pregnancy and the postpartum period ".  (see lines 508-512) |
|  | 2. Kindly indicate results about the participants' demography both in the abstract and main text? | Thank you for your insightful feedback. In the revised main manuscript section, we have taken into account the comments from the first round of reviewers and adjusted the description of demographic characteristics from the results section to the participants section. However, as you rightly pointed out, we need to further enhance the presentation of this content to provide other researchers with a clearer understanding of the study's research subjects. In response to your suggestions, we have included additional details regarding demographic characteristics in the summary section, such as the average age, parity, registered residence of participants, the average number of community participations, and the average mental health score. Furthermore, we have added a table of demographic characteristics based on the existing data in the participants section. (see lines 231-237 , and Table 1) |
|  | 3. Please confirm that all typographical errors have been corrected. Check lines 121-122: "the rise of the social media and social media…" | Thank you for your rigorous attention to linguistic details. In response to your comments, we carefully revised each of the issues you pointed out and conducted a thorough language review of the entire manuscript to improve the clarity and professionalism of our academic writing. |
|  | 4. Indicate if the survey questionnaire, the 'Maternal Depression Generation Trajectory and Social Support Research,' had been pretested prior to the study. Or was it originally designed for this study? You mentioned the source of some survey instruments used, please do the same for all instruments. | Thank you for your insightful comment. Based on your feedback, we would like to provide further clarification regarding the “Maternal Depression Generation Trajectory and Social Support Research” questionnaire. We appreciate the opportunity to elaborate on this point and hope the following explanation addresses your concerns.  Questionnaire Design. The questionnaire was originally designed for this study, based on a specific theoretical framework and research questions concerning the generative mechanisms of maternal depression and its relationship to social support.  Pretesting. The present study employs a survey questionnaire as a method for data collection. Prior to executing the pretest, the questionnaire was finalized, and a pretest form was prepared using the framework from Hu, S. (2023)[1]. The survey questionnaire was administered face-to-face in the hospital to facilitate the distribution of both the questionnaire and the pretest form. The stages undertaken during the pretest are as follows:  1. Selecting the Instrument: The researcher clearly defined each construct within the framework in a manner consistent with past research. This approach was informed by a literature review of previous theoretical and empirical studies on the constructs, as recommended.  2. Expert Review for Validity: This stage was conducted prior to the distribution of the survey questionnaire to ensure the validity of the instruments. The researcher selected and consulted three experts in the field of maternal public mental health; among them, there was one midwife, one mental health professional, and one social medical expert.  3. Application for Ethics Committee Approval: The researcher applied for ethics committee approval before the data collection phase.  4. Selecting Respondents: At this stage, the researchers recruited 30 pregnant women to participate in the questionnaire survey at the hospital with the highest number of births per year.  5. Executing the pretest. The questionnaire was distributed face-to-face in this prediction test by researchers to participants, and was subsequently collected after all participants had completed their responses. The prediction test form consists of three parts: the first part is the cover letter, the second part is the questionnaire, and the third part is the prediction test form. The cover letter briefly introduces the research background, the purpose of the prediction test, the implementation process, and guarantees the anonymity and confidentiality of the respondents. The second part of the questionnaire contains specific topics related to each variable, while the prediction test requests respondents to provide feedback on the questions and scales in the form of queries and to suggest improvements.  6. Review results. SPSS software was employed to assess the validity and reliability of the collected data. It was determined that the items in the instrument met the minimum acceptable requirement of a Cronbach's alpha of 0.7. Feedback from participants regarding unclear terminology and ambiguous expressions in individual items was addressed and corrected.  Source of Instruments. As you rightly pointed out, some scales or items were adapted from existing validated instruments. Below is a complete list of all instruments used, along with their sources: (1) Mother's PMH. This questionnaire was utilized to assess the mental health of Chinese mothers, specifically the revised Body Mind-Spirit Well-Being Inventory (BMSWBI) [2]. (2) Community Participation. This variable follows Lin's research methodology to assess community connections through involvement in community organizations [3]. (3) Social Support from Peers. This questionnaire measured the support received from offline peer groups, specifically pregnant women and social media, utilizing the framework from Olson et al. [4]. (4) Partners’ Communication. The Initiator Style Questionnaire (ISQ), developed by Denton and Burleson [5], prompts mothers to assess their inclination towards initiating or avoiding discussions about relationship issues with their partners.  In response to your comment, we have revised the manuscript to clearly indicate the origin of each instrument and noted the pretest process in the methods section. We have added this information to the methods section of the manuscript. (see lines 189-196, and references 16,38,40-41) |
|  | 5. Indicate how long it took to complete the survey. | Thank you for your valuable comment. We have included the complete duration for the survey in the methods section of the manuscript. Specifically, we state: "On average, participants took approximately 20-30 minutes to complete the survey. This duration was recorded during the pretest phase and reconfirmed during the formal data collection process". (see lines 204-205) |
|  | 6. Please report the challenges that were encountered in the course of the study. | Thank you for your valuable comment. As you have rightly pointed out, we encountered several challenges during the investigation process. However, due to the manuscript's word limit, we were unable to elaborate on these details in the main text. We sincerely appreciate your understanding and would like to provide a clearer explanation in this document.  Challenges 1 Participant Recruitment. Given the sensitive nature of maternal mental health, some potential participants were initially hesitant to engage in the survey. To address this issue, we adopted a two-pronged approach. First, we displayed posters in the obstetrics clinics of each participating hospital to introduce the purpose and significance of the study, thereby promoting transparency and ensuring informed consent. Second, we distributed the questionnaires during the waiting periods for routine prenatal check-ups, a time when participants were relatively at ease and more likely to have the time and willingness to thoughtfully complete the survey. This approach helped reduce refusal rates and improved the quality and completeness of the data collected.  Challenges 2 Data Collection in Clinical Settings. Conducting research within hospital settings posed a significant challenge due to the requirement for multi-level administrative approvals to access specific departments. To overcome this barrier, we established formal collaborations with the research administration offices of each participating hospital. These institutional partnerships not only expedited the approval process but also enhanced the study's credibility among clinical staff. Consequently, we were able to build trust with healthcare professionals, which facilitated smoother and more efficient data collection.  Challenges 3 COVID-19 Related Disruptions. Intermittent restrictions during the pandemic delayed in-person visits and necessitated adjustments in recruitment and scheduling. The investigation coincided with the COVID-19 epidemic, which required adherence to China's epidemic prevention policies. Upon identifying a suspected case, quarantine measures were implemented. During the investigation, a suspected case emerged in the obstetrics inpatient departments of Hospital B, where we were conducting our research. Consequently, all obstetric medical staff, pregnant women, and some researchers were transferred, forcing the research team to halt the obstetrics investigation for two months. This experience of navigating the COVID-19 epidemic alongside the obstetric medical team and pregnant women has, to some extent, strengthened our trust and facilitated further research and investigation.  Thank you for your guidance, which will assist us in further optimizing and enhancing the content of the limitations sections. Due to the word limit of the main manuscript, we have included some of the content from the challenges in the limitations section. (see lines 494-514) |
|  | 7. Did you consider if parity could have mediated any of the findings? Did support differ by parity? e.g. did first time mothers have more support than second or third timers? | Thank you for raising an important point regarding parity as a potential factor of study findings, particularly concerning the differences in support received by first-time versus experienced mothers. To address this, we conducted a one-way ANOVA comparing indicators of psychological well-being and social support across three parity groups, first-time mothers, second-time mothers, and those with three or more pregnancies.  Our analysis revealed that most psychological indicators—such as maternal positive mental health (PMH), body, mind, spirit, and informational support—did not differ significantly by parity (all *p* > .05). However, several key support-related outcomes exhibited significant differences. Specifically, first-time mothers reported significantly higher levels of community participation (F = 13.56, *p* < .001), social support (F = 13.46, *p* < .001), emotional support (F = 12.92, *p* < .001), and instrumental support (F = 26.81, *p* < .001) compared to those with previous pregnancies. Post-hoc analyses (with LSD) indicated small to moderate effect sizes between first-time and multiparous mothers; for instance, instrumental support was notably higher among first-time mothers compared to both second-time (d = 0.337*) and third-or-more-time mothers (d = 0.538*). These findings suggest that first-time mothers may benefit from heightened levels of social attention and support. In contrast, mothers with multiple pregnancies may receive relatively less support, which could have implications for designing targeted interventions.  We sincerely appreciate your suggestion once again, as this analysis offers valuable insights into how parity status may influence maternal support experiences. Given the word limit constraints of the main manuscript, we have summarized these parity-based findings in a supplementary table and briefly noted them in the "Correlation analysis between the main variables" section.  (see lines 337-341 in the manuscript, and Appendix 2 at the end of this document) |
|  | 8. Since participants were recruited from 3 different facilities, did your findings differ based on location or was the analysis aggregated? It would be interesting to see the differences and similarities by location. | Thank you for raising an important point regarding facilities as a potential factor influencing study findings, particularly concerning the differences in support received by the three hospitals, DH-A, DH-B, and DH-C. To address this, we conducted a one-way ANOVA to compare key PMH and support-related variables across these three facilities, followed by post-hoc comparisons to further investigate specific group differences.  Our analysis revealed that mother's PMH, mind, spirit, social support, emotional support, and informational support did not show statistically significant differences across hospitals (all *p* > .05). This suggests a relative consistency in these domains across locations. In contrast, body-related well-being (F = 3.89, *p* = .02) and instrumental support (F = 3.10, *p* = .05) exhibited modest but significant differences by location. Post-hoc analyses (with LSD) indicated that DH-C participants reported higher levels in both indicators compared to DH-A and DH-B (e.g., body: DH-C > DH-B, d = 0.145*, DH-C > DH-A, d = 0.176*; instrumental support: DH-C > DH-A, d = -0.169*). Notably, community participation demonstrated the most marked difference across hospitals (F = 9.70, *p* < .001), with participants at DH-C reporting significantly greater involvement than those at DH-A (d = -0.662*) and DH-B (d = -0.286). These differences may reflect variations in outreach programs at C hospital or regional community engagement levels.Overall, while the majority of psychosocial indicators were relatively consistent across sites, a few context-sensitive variables, such as community participation and instrumental support, exhibited statistically significant location-based variation. These findings suggest that while aggregated analysis was appropriate for many variables, site-specific differences should be noted and may warrant further exploration in future research.  We sincerely appreciate your suggestions once again, as this analysis provides valuable insights into how the status of different facilities may influence maternal support experiences. Given the word limit constraints of the main manuscript, we have summarized three facility-based findings in a supplementary table and briefly noted them in the "Correlation Analysis Between the Main Variables" section.  (see lines 333-337 in the manuscript and Appendix 3 at the end of this document) |
|  | 9. Are the study sites urban or rural? Don’t you think this could have influenced the availability, type and quality of support available to pregnant women? | Thank you for highlighting the critical issue of the urban-rural background of the research site and its potential impact on the availability, type, and quality of support for pregnant women.The three hospitals involved in the study are situated in urban areas of Jiangsu Province. However, Jiangsu is a highly urbanized region that has seen a substantial influx of rural populations migrating to cities, particularly in the surveyed city of Wuxi (WX), which experiences a significant floating population. According to statistical data, the permanent population of the surveyed cities is 7.505 million, with an urbanization rate of 86.61%. This includes a registered population of 5.2294 million and a non-registered population of 2.2756 million.Consequently, the obstetrics wards of these hospitals cater to diverse populations, including urban residents, rural residents, and rural women from other provinces who reside in the city for work or education. To more accurately reflect this diversity, our research employs registered residence status as the analytical variable, rather than relying solely on the binary rural/urban classification. Among the 1,899 participants, 975 had Wuxi household registration (local residents), while 730 were non-locals, typically from rural or suburban areas. Previous research, along with our findings, indicates that household registration status more effectively reflects disparities in access to resources, institutional support, and social networks than the traditional rural/urban dichotomy. For instance, local residents are more likely than non-local residents to receive family support and welfare services in cities where they have registered residence.  Based on your feedback, we conducted a one-way ANOVA to compare key positive mental health (PMH) and support-related variables across different resident groups, followed by post-hoc comparisons to further investigate specific group differences.Local residents reported significantly higher scores in positive mental health (PMH) (F = 13.56, p < .001), mental well-being (mind: F = 21.88, p < .001), and spiritual well-being (spirit: F = 13.77, p < .001) compared to non-local participants. Significant differences also emerged in support dimensions; local mothers reported higher levels of social support (F = 35.09, p < .001), emotional support (F = 11.55, p < .001), informational support (F = 19.79, p < .001), and instrumental support (F = 31.25, p < .001). No significant differences were observed in body-related well-being (F = 0.18, p = .67) or community participation (F = 0.84, p = .36).These findings suggest that non-local pregnant women—who may face greater mobility challenges, reduced access to extended family, and limited familiarity with local resources—tend to report lower levels of support and mental well-being. We sincerely appreciate your suggestions once again, as this analysis provides valuable insights into how urban versus rural status may influence maternal support experiences. Given the word limit constraints of the main manuscript, we have summarized the findings related to different residents in a supplementary table and briefly noted them in the "correlation analysis between the main variables" section and discussion section.  (see lines 330-332, 456-459, in the manuscript and Appendix 4 at the end of this document) |
|  | 10. What type of incentives/small gift was provided for the participants? When was this provided? Before or after filling out the survey? You should detail this to show that undue inducement was avoided. | Thank you for your thoughtful comment regarding participant incentives and ethical considerations. To express appreciation for participants’ time and contribution, we provided each respondent with a small token gift valued at approximately 10 RMB (e.g., a pack of tissues or a handheld fan). This item was selected for its low monetary value and practical relevance, in line with ethical guidelines to avoid any form of undue inducement.Importantly, the incentive was provided only after the participant completed the survey, and only after they had voluntarily agreed to participate via the informed consent process. At no point was the gift used as a condition or persuasion to recruit participants. We have clarified this procedure in the revised methods section of the manuscript , and we confirm that the incentive plan was also approved as part of the study’s ethical review process. |
|  | 11. You indicated that "Eligible participants were guided by the research team to view the online research information form and consent form…" You also need to explain the mode of instrument administration. Was it by the researcher, participants or by who? This is important for replicability. | Thank you for your insightful comment. After eligible participants were guided by the research team to review the online research information sheet and provide informed consent, a paper-based questionnaire was distributed to each participant by the researcher. Participants completed the questionnaire independently on-site and returned it to the research team upon completion. The completed paper questionnaires were then entered into the Credamo online survey platform by the research team. A two-round verification process was conducted by research supervisors to ensure the accuracy and consistency of data entry. |
|  | 12. Describe the rationale for the sample size selection. What method of sampling was utilized? Was it based on population size or what? How did you arrive at the total (1899) | Thank you for the questions and valuable suggestions on sampling methods.  In this study, power analysis and quota sampling methods were used for sample sampling and sample size determination. The detailed sample size selection process is as follows:  To ensure the adequacy of the sample for statistical analysis, we first conducted a statistical power analysis using G*Power software. The analysis was based on a medium effect size (f² = 0.15), with parameters set at α = 0.05, power (1 − β) = 0.95, and a two-tailed test. The results indicated that a minimum of 107 participants would be required to detect statistically significant effects in the proposed model. Given this, the final sample size used in the study exceeds the required minimum, demonstrating that the study possesses sufficient statistical power to test the research hypotheses.  After determining the minimum threshold, we employed a quota sampling strategy, complemented by collaborative sampling through partner hospitals[6, 7]. This approach was employed not only to meet the statistical requirements but also to maximize the representativeness and diversity of the sample across various demographic and social backgrounds.The number of births per year at Hospital A is approximately 10,000, while Hospital B has over 5,300, and Hospital C nearly 5,100. We utilized a cross-quota sampling method that combined two characteristics of the surveyed hospitals and the gestational period. With the proportion of pregnancies balanced (50/50), the sampling ratio of survey locations was set at 10/100. A total of 2,030 individuals were sampled, including 1,000 participants from Hospital A, comprising 500 mothers in early pregnancy (12 to 28 weeks) and 500 mothers in later pregnancy (more than 28 weeks). Additionally, 530 participants were drawn from Hospital B, with 260 mothers in early pregnancy and 270 mothers in later pregnancy. Lastly, 500 participants were sampled from Hospital C, consisting of 250 mothers in early pregnancy and 250 mothers in later pregnancy.  Ultimately, during the collaborative investigation, we collected 948 cases from Hospital A, 487 cases from Hospital B, and 464 cases from Hospital C, resulting in a total of 1,899 responses. This multi-site, stage-stratified sampling design ensured a balanced representation of maternal experiences across different healthcare settings and pregnancy stages, thereby supporting the robustness and generalizability of the study findings.  Following your comments, we provide a detailed description of sample size selection and sampling approach in the Methods section and insert citations of relevant literature.  (see lines 219-231, and Appendix 1 at the end of this document) |
|  | 13. You should add a separate section for limitations of your study rather than its current location within the discussion. Add other limitations to what is already reported. E.g., the study could have benefited from triangulation of methods and participants. 1) only pregnant women were sampled without considering the perspectives of their social networks, such as partners. 2) Also only survey was used in the study, using a mixed methodology could have enriched the findings further, etc. | Thank you for this helpful suggestion. In response, we have created a dedicated “Limitations” section in the revised manuscript, separating it from the main discussion to enhance clarity and transparency.We appreciate your guidance, which helped us strengthen the methodological transparency and critical reflection within the paper.In addition to the previously noted limitations, we have now included the following points as per your valuable recommendation.  First, the study relied solely on self-reported data from Chinese pregnant women, omitting perspectives from partners, family members, or healthcare providers. This limitation may affect the generalizability of the findings and overlook important social dynamics. To address this issue, a follow-up qualitative study involving key members of the social network has been conducted and will be reported separately.  Secondly, although the Body–Mind–Spirit Well-Being Inventory was selected for its cultural relevance, it does not evaluate clinical symptoms such as depression or anxiety. The lack of standardized diagnostic tools (e.g., PHQ-9, GAD-7) may hinder clinical interpretation. Future studies should consider integrating both culturally grounded and clinical measures.  Thirdly, the COVID-19 quarantine policy has significantly hindered our ability to collect cross-regional and large-scale data. Consequently, this study utilized regional samples and gathered data in clinical settings, which may limit our capacity to observe the evolution of mental health and support throughout pregnancy and the postpartum period. To address this limitation, the research team is currently conducting a longitudinal qualitative study that tracks 83 couples throughout pregnancy and postpartum to explore changes in mental health and support over time.(see lines 494-514) |
| **Reviewer 2** | 1. Authors would need to upload the anonymized datafile as a supplementary material in order for the current statement to be correct. My understanding from last review is that there were restrictions for the data. If this is still the reason for being unable to share data then I would recommend for authors to use the statement below instead (adding an email address to the ethics committee and not the author), or an alternative wording recommended by the editor:"Data cannot be shared publicly because of data contain potentially identifying and sensitive patient information. Data are available from the Medical Ethics Committee of Jiangnan University   (JNU20211217IRB01) (contact via INSERT EMAIL) for researchers who meet the criteria for access to confidential data." | Thank you for your attention to the data availability statement. You are correct due to ethical restrictions and the presence of potentially identifying and sensitive patient information; the dataset cannot be shared publicly. In line with your recommendation, we have revised the data availability statement in the manuscript as follows.  We recognize the significance of data sharing in promoting research transparency and replicability. However, due to the sensitivity of the data and our ethical commitment to the research participants, we have opted to impose certain limitations. The raw data we analyzed contains potentially identifiable and sensitive maternal information, even when encoded. In accordance with the ethics agreement approved by the ethics committee, we ensure the anonymity of participants and the confidentiality of the data. Access to the data is available through the Medical Ethics Committee of Jiangnan University (JNU20211217IRB01) (zhouyizhou@jiangnan.edu.cn) for researchers who meet the criteria for accessing confidential data. Although we are unable to share the original interview data, we believe that the methods and tools employed guarantee the repeatability of our research. Therefore, we have included a detailed introduction to the data collection and analysis processes in the research methods section to equip other researchers with the necessary information.  （see lines 545-558） |

**References**

[1] Hu S. Pretesting. In: Maggino F, editor. Encyclopedia of Quality of Life and Well-Being Research. Cham: Springer International Publishing; 2023. p. 5452-6.
[2] Ng S, Yau JK, Chan CL, Chan CH, Ho DY. The measurement of body-mind-spirit well-being: Toward multidimensionality and transcultural applicability. Social work in health care. 2005;41(1):33-52.
[3] Lin N, Ye X, Ensel WM. Social support and depressed mood: A structural analysis. Journal of Health and Social behavior. 1999:344-59.
[4] Olson DA, Shultz KS, Gender differences in the dimensionality of social support. J. Appl. Soc. Psychol. 1994; 24 (14): 1221-1232.
[5] Denton WH, Burleson BR. The Initiator Style Questionnaire: A scale to assess initiator tendency in couples. Personal Relationships. 2007;14(2):245-68.
[6] American public opinion in the 1930s and 1940s: The analysis of quota-controlled sample survey data. International Journal of Public Opinion Quarterly. 2006; 70:499–529.
[7] Couper MP, Dever JA, Gile KJ. Report of the AAPOR task force on non-probability sampling. Retrieved November. 2013; 8:2020.

Appendix 1 Proportion of cross-quotas in the sample

| Gestation period | Hospital | | | Total |
| --- | --- | --- | --- | --- |
|  | A | B | C |  |
| Early pregnancy (12 to 28 weeks) | 500 | 260 | 250 | 1010 |
| Later pregnancy (28 to 41 weeks) | 500 | 270 | 250 | 1020 |
| Total | 1000 | 530 | 500 | 2030 |
|  |  |  |  |  |

Appendix 2 Descriptive and inferential statistics for the diagnostics of main variables categorized by different hospitals

| Predictors | Study sample | Different Hosptials (DH) | | | ANOVA | | Post-hoc-Analyses | | |
| --- | --- | --- | --- | --- | --- | --- | --- | --- | --- |
|  |  | DH-A | DH-B | DH-C |  |  | DH-A vs. DH-B | DH-A vs. DH-C | DH-B vs. DH-C |
|  | *M±SD(N)* | *M±SD(N)* | | | F | p | d | | |
| Mother's PMH | 4.23±0.74 (1705) | 4.24±0.73 (775) | 4.27±0.75 (453) | 4.17±0.74 (477) | 2.48 | 0.08 | -0.025 | 0.077 | 0.102* |
| Body | 4.07±1.11 (1705) | 4.12±1.10 (775) | 4.10±1.13 (453) | 3.95±1.11 (477) | 3.89 | 0.02 | 0.023 | 0.176* | 0.145* |
| Mind | 4.51±0.99 (1705) | 4.50±1.02 (775) | 4.54±0.96 (453) | 4.49±0.96 (477) | 0.36 | 0.70 | -0.041 | 0.010 | 0.051 |
| spirit | 4.12±0.82 (1705) | 4.12±0.82 (775) | 4.17±0.84 (453) | 4.07±0.81 (477) | 1.80 | 0.17 | -0.053 | 0.049 | 0.100 |
| Community participation | 4.43±2.65 (1705) | 4.14±2.60 (775) | 4.52±2.70 (453) | 4.81±2.62 (477) | 9.70 | 0.00 | -0.376* | -0.662* | -0.286 |
| Social support | 4.47±0.85 (1705) | 4.44±0.86 (775) | 4.46±0.85 (453) | 4.53±0.82 (477) | 1.51 | 0.22 | -0.017 | -0.084 | -0.067 |
| Emotional support | 4.65±0.91 (1705) | 4.62±0.93 (775) | 4.68±0.88 (453) | 4.69±0.89 (477) | 1.03 | 0.36 | -0.058 | -0.067 | -0.009 |
| Informational support | 4.07±1.11 (1705) | 4.07±1.11 (775) | 4.02±1.15 (453) | 4.10±1.09 (477) | 0.71 | 0.49 | 0.055 | -0.030 | -0.085 |
| Instrumental support | 4.70±1.17 (1705) | 4.64±1.22 (775) | 4.69±1.15 (453) | 4.81±1.10 (477) | 3.10 | 0.05 | -0.056 | -0.169* | -0.113 |

Appendix 3 Descriptive and inferential statistics for the diagnostics of main variables based on the number of pregnancies

| Predictors | Study sample | Pregnant times | | | ANOVA | | Post-hoc-Analyses | | |
| --- | --- | --- | --- | --- | --- | --- | --- | --- | --- |
|  |  | 1st | 2nd | 3rd  or more |  |  | 1st vs. 2nd | 1st vs. 3rd | 2nd vs. 3rd |
|  | *M±SD(N)* | *M±SD(N)* | | | F | p | d | | |
| Mother's PMH | 4.23±0.74 (1700) | 4.22±0.73 (1000) | 4.28±0.76 (487) | 4.15±0.74 (213) | 2.27 | 0.10 | -0.055 | 0.072 | 0.127 |
| Body | 4.07±1.11 (1700) | 4.07±1.11 (1000) | 4.11±1.12 (487) | 3.99±1.13 (213) | 0.87 | 0.42 | -0.042 | 0.078 | 0.119 |
| Mind | 4.51±0.99 (1700) | 4.50±0.98 (1000) | 4.58±1.00 (487) | 4.44±1.00 (213) | 1.81 | 0.16 | -0.081 | 0.058 | 0.139 |
| spirit | 4.12±0.82 (1700) | 4.12±0.82 (1000) | 4.16±0.81 (487) | 4.04±0.85 (213) | 1.61 | 0.20 | -0.043 | 0.078 | 0.120 |
| Community participation | 4.43±2.65 (1700) | 4.70±2.65 (1000) | 4.11±2.54 (487) | 3.87±2.71 (213) | 13.56 | 0.00 | 0.585* | 0.827* | 0.242 |
| Social support | 4.47±0.85 (1700) | 4.55±0.80 (1000) | 4.39±0.88 (487) | 4.27±0.93 (213) | 13.46 | 0.00 | 0.168* | 0.284* | 0.116 |
| Emotional support | 4.65±0.91 (1700) | 4.62±0.93 (1000) | 4.68±0.88 (487) | 4.69±0.89 (213) | 12.92 | 0.00 | 0.177* | 0.298* | 0.121 |
| Informational support | 4.07±1.11 (1700) | 4.09±1.10 (1000) | 4.04±1.14 (487) | 4.01±1.16 (213) | 0.71 | 0.49 | 0.054 | 0.084 | 0.030 |
| Instrumental support | 4.70±1.17 (1700) | 4.86±1.94 (1000) | 4.53±1.27 (487) | 4.32±1.35 (213) | 26.81 | 0.00 | 0.337* | 0.538* | 0.201 |

Appendix 4 Descriptive and inferential statistics for the diagnostics of main variables by household registration certificates

| Predictors | Study sample | Local residents | | ANOVA | |
| --- | --- | --- | --- | --- | --- |
|  |  | Nonlocal | Local |  |  |
|  | *M±SD(N)* | *M±SD(N)* | | F | p |
| Mother's PMH | 4.23±0.74 (1705) | 4.15±0.77 (730) | 4.29±0.71 (975) | 13.56 | 0.00 |
| Body | 4.07±1.11 (1705) | 4.06±1.14 (730) | 4.08±1.09 (975) | 0.18 | 0.67 |
| Mind | 4.51±0.99 (1705) | 4.38±1.00 (730) | 4.61±0.96 (975) | 21.88 | 0.00 |
| spirit | 4.12±0.82 (1705) | 4.04±0.84 (730) | 4.19±0.80 (975) | 13.77 | 0.00 |
| Community participation | 4.43±2.65 (1705) | 4.36±2.80 (730) | 4.48±2.53 (975) | 0.84 | 0.36 |
| Social support | 4.47±0.85 (1705) | 4.33±0.89 (730) | 4.57±0.80 (975) | 35.09 | 0.00 |
| Emotional support | 4.65±0.91 (1705) | 4.57±0.97 (730) | 4.72±0.85 (975) | 11.55 | 0.00 |
| Informational support | 4.07±1.11 (1705) | 3.93±1.16 (730) | 4.17±1.06 (975) | 19.79 | 0.00 |
| Instrumental support | 4.70±1.17 (1705) | 4.52±1.23 (730) | 4.83±1.10 (975) | 31.25 | 0.00 |
